# EGP-Net: a lung nodule segmentation network integrating edge guidance and pyramidal multi-scale contextual attention mechanisms

**DOI:** 10.3389/fonc.2026.1875891

**Published:** 2026-07-02

**Authors:** Xiangsuo Fan, Lihong Deng, Jiachen Hou, Tao Li, Zhougui Ling, Shuping Li

**Affiliations:** 1 School of Automation, Guangxi University of Science and Technology, Liuzhou, China; 2 Earthwork Collaborative Innovation Center, Guangxi University of Science and Technology, Liuzhou, China; 3 Department of Medical Imaging, The Fourth Affiliated Hospital of Guangxi Medical University, Liuzhou, Guangxi, China; 4 Department of Medical Imaging, Liuzhou Worker’s Hospital, Liuzhou, Guangxi, China

**Keywords:** edge guidance, feature fusion, hybrid loss function, lung nodule segmentation, medical image segmentation

## Abstract

**Objectives:**

Accurate segmentation of pulmonary nodules in CT images is of great significance for the early screening, diagnosis, and treatment planning of lung cancer. However, manual segmentation is time-consuming and highly subjective, and existing pulmonary nodule segmentation methods still struggle to achieve accurate segmentation under challenging conditions such as blurred boundaries and interference from complex structures. This study aims to develop a segmentation method for pulmonary nodules to improve segmentation accuracy and support the clinical evaluation of lung cancer.

**Materials and methods:**

The proposed method, EGP-Net, integrates a Res2Net-50 encoder, an edge-guided network, a global pyramid perception module, a dynamic attention fusion module, and a multi-scale contextual decoder. By combining contextual information with boundary-aware features, the network can effectively represent pulmonary nodules. The model was trained and evaluated on the public LIDC dataset and a private clinical dataset, and segmentation performance was assessed using IoU, Dice, F2-score, and F0.5-score.

**Results:**

On the LIDC dataset, EGP-Net achieved an IoU of 88.32% and a Dice coefficient of 92.65%, outperforming state-of-the-art comparative segmentation methods. It also achieved excellent performance on the private clinical dataset. Ablation experiments further verified the effectiveness of each component.

**Conclusions:**

EGP-Net improves the accuracy and robustness of pulmonary nodule segmentation, facilitating precise nodule identification and quantitative analysis, and providing reliable support for lung cancer detection and evaluation.

## 1 Introduction

Lung cancer is a malignant disease with persistently high incidence and mortality rates. Early symptoms of lung cancer often manifest as pulmonary nodules, and the early detection of these nodules can improve the cure rate of patients with lung cancer. Different types of pulmonary nodules presenting with different symptoms. Therefore, early detection of pulmonary nodules facilitates timely identification of potential malignant lesions.

Currently, medical imaging technologies such as Nuclear Magnetic Resonance Imaging (NMRI) and Computed Tomography(CT) are widely used for early lung cancer detection. In particular, low-dose CT has been extensively applied in clinical practice due to its advantages of low radiation, high resolution, and rapid imaging. Accurate segmentation of pulmonary nodules helps delineate their boundaries, thereby assisting clinical diagnosis and treatment planning (1, 2). However, the large number of CT slices makes manual annotation time-consuming and labor-intensive, which has driven the development of computer-aided detection (CAD) systems. Nevertheless, automatic segmentation of pulmonary nodules remains challenging due to significant variations in size, irregular shapes, and low contrast with surrounding tissues in CT images. To address these challenges, researchers have proposed various segmentation methods, including both traditional approaches and deep learning-based methods, achieving significant progress.

Traditional segmentation methods, such as threshold-based segmentation algorithms, region growing algorithms, edge detection algorithms, active contour models, iterative local thresholding segmentation algorithms, and clustering methods Fazilov et al. (3)Shiny and Sugitha (4)Sheela and Suganthi (5)Akbari et al. (6), primarily involve preprocessing operations such as converting images to grayscale, applying filtering techniques, and performing binarization. This approach reduces errors and improves similarity metrics; however, traditional methods still suffer from edge loss and misclassification due to low contrast and blurred boundaries, which hinder precise delineation and diagnosis.

In recent years, deep learning-based methods have emerged as mainstream approaches. By leveraging encoder-decoder architectures and backpropagation algorithms, these methods can automatically learn image features without human intervention, achieving higher segmentation accuracy, robustness, and generalization capabilities. Deep learning-based medical image segmentation methods have achieved significant advancements across various domains, particularly the application of convolutional neural networks (CNN) for medical image segmentation (7–11). For example, UNetRonneberger et al. (12) and its variants have achieved remarkable performance in various medical image segmentation tasks due to their encoder–decoder architecture and skip connections. Accordingly, researchers have proposed various deep learning models for lung nodule segmentation. For instance, Banu et al. (13) proposed AWEU-Net, which enhances key feature representation by introducing spatial and channel attention mechanisms, thereby improving feature fusion. Tang et al. Tang et al. (14) introduced the SM-RNet, which combines multiple attention mechanisms and scale awareness. Employing a progressive “backward erasure” strategy enhanced the segmentation performance of lung nodules. Wang et al. (15) proposed UCTransNet, which replaces U-Net’s skip connections with transformers to reduce the semantic gap between the encoder and decoder. Cai et al. (16) developed an MDFN network by utilizing self-calibrated edge enhancement techniques and dynamically fusing features across multiple levels to better handle the fine-grained edge details of lung nodules.

Although the aforementioned studies have achieved considerable progress in automatic pulmonary nodule segmentation, several challenges remain. First, the direct skip connections between the encoder and decoder tend to weaken deep semantic information and introduce redundant features, leading to insufficient semantic representation. Second, pulmonary nodule boundaries are often blurred and connected to structures such as blood vessels and bronchi, making it crucial to effectively exploit boundary information from low-level features while suppressing background interference. Third, simple linear fusion strategies fail to capture the complex relationships between global semantic information and local boundary features, limiting effective multi-level feature integration. Therefore, effectively leveraging transfer learning features and designing specialized segmentation networks are essential for accurate pulmonary nodule segmentation.

To this end, this paper proposes a 2D lung nodule segmentation network that integrates edge guidance and pyramidal multi-Scale contextual attention mechanisms (EGP-Net). This network comprises an encoder network, an edge-guided network, a global pyramid perception module (GPP), a dynamic attention fusion module(DAF), and a multi-scale context decoder network. Outputs from encoder stages 1 to 4 are fed into the GPP module to reconstruct and guide cross-layer global semantic flow. Concurrently, low-level encoder features were integrated into the edge guidance network to extract fine-grained boundary information. The GPP module selectively guides deep global semantic information back to shallow layers, mitigating the semantic weakening and gaps inherent in traditional skip connections. The DAF performs adaptive weighted fusion of features across different semantic levels, reducing information redundancy caused by simple linear operations, thereby improving the overall segmentation performance. In addition, a multi-scale context decoder network was designed to reconstruct deep semantic information through sub-pixel convolutional operations on global context information guided by the GPP module.

The main contributions of this paper are summarized below:

1. We design an edge-guided network that fully leverages the fine-grained information embedded in low-level features of the encoder to achieve precise modeling of boundary details. This network effectively suppresses interference from background structures such as blood vessels and bronchi, enabling the model to more accurately localize nodule boundaries.
2. We propose a Global Pyramid Perception module, which works in conjunction with a multiscale context decoder. By fusing multi-level semantic information and combining it with sub-pixel convolution upsampling, the module achieves precise feature reconstruction, effectively mitigating scale variation and reducing upsampling blurring.
3. We design a Dynamic Attention Fusion module that adopts a dual-path architecture integrating Grouped Attention Gating and Dual-branch Hybrid Attention Fusion, enabling adaptive weighting of heterogeneous features, enhancing salient regions, and ultimately improving segmentation performance.

## 2 Related work

This section briefly outlines three categories of research that are most relevant to the proposed lung nodule segmentation method: 2.1 automatic medical image segmentation, 2.2 multi-scale feature extraction, and 2.3 boundary-based segmentation models.

### 2.1 Automated medical image segmentation

In recent years, deep learning has achieved substantial progress in medical image segmentation. Ronneberger et al. (12) proposed U-Net, which integrates shallow spatial features with deep semantic information through skip connections, where direct feature concatenation may introduce inconsistencies across different semantic levels. Chen et al. (17) introduced Transformers into U-Net to enhance global context modeling and improve multi-organ and tumor segmentation. However, it may perform unstably on small-sample or high resolution tasks. Sun et al. (18) report that DA-TransUNet enhances the capture of global context and fine-grained details through Transformers and DA-Blocks, but it also introduces increased model complexity, a decoder that is not specifically optimized, and a risk of losing fine-grained information during encoder tokenization. Bruschi et al. (19) proposed U-Grad, which integrates Grad-CAM-guided attention with a reduced U-Net architecture to enhance lesion-focused feature learning and improve computational efficiency. Lan et al. (20) proposed BRAU-Net++, which leverages bi-level routing attention and the SCCSA module to enhance global context modeling and multi-scale feature interactions. However, the model suffers from high complexity and relies on specific attention mechanisms. Rezvani et al. (21) proposed FusionLungNet, which employs channel-wise aggregation attention and multi-scale feature fusion with refinement modules to improve contextual modeling and segmentation accuracy. Therefore, it is critical to design segmentation architectures that efficiently optimize cross-layer semantic consistency and facilitate feature interaction.

### 2.2 Multi-scale feature extraction

Multi-scale feature extraction plays a crucial role in improving segmentation performance by modeling targets with varying sizes and shapes. Fu et al. (22) proposed HmsU-Net, which combines Transformers and CNNs to capture multi-scale contextual information, with multiscale modeling primarily centered on feature aggregation and limited explicit semantic guidance from deeper layers to shallow representations. Hua et al. (23) introduced a multiscale and multi-view feature interaction module to enhance feature representation across different resolutions, where feature interaction is mainly performed within similar semantic levels. Guo et al. (24) developed Res2-CD-UNet to enhance multi-scale feature representation through Res2Net blocks and ConvFormer, while the network still suffers from considerable architectural complexity and computational overhead. Xu et al. (25) proposed a multi-scale attention-based framework using atrous convolutions and collaborative attention for feature extraction and weighting. However, the fusion of attention and multi-scale features is relatively simple and lacks deep joint interaction. To address the limitations of existing multi-scale methods, we introduce a global pyramid perception module that explicitly back propagates deep semantic context through multi-layer skip connections, thereby enhancing semantic alignment and achieving more efficient multi-scale feature fusion.

### 2.3 Boundary segmentation model-based approach

Boundary information has been widely exploited in medical image segmentation to improve contour localization and structural delineation. Sun et al. (26) proposed a Boundary DoU loss for medical image segmentation that reformulates boundary IoU into a differentiable objective using intersection–difference decomposition, with an adaptive *α* strategy to balance boundary emphasis across different target sizes. Lin et al. (27) proposed a hybrid architecture combining CNNs, Vision Transformers, and edge detection operators to improve segmentation accuracy, where boundary learning and semantic representation are modeled in a relatively decoupled manner. Yang et al. (28) introduced a multi-task bidirectional boundary-aware network to encode forward and backward boundary information, in which boundary supervision is applied in parallel and exhibits limited interaction with multi-scale semantic features. Yu et al. (29) proposed BG-Net with a gradient convolution module and a boundary-aware GC branch to enhance edge feature extraction, where directional and cyclic gradient operators are used to improve local boundary sensitivity and are further integrated with a U-shaped backbone for global feature representation and multimodal fusion. Motivated by these limitations, our method incorporates an edge-aware and semantically guided feature fusion strategy that tightly couples boundary cues with hierarchical semantic representations, thereby improving both boundary delineation and overall segmentation performance. **Table 1** presents a summary and comparative analysis of related studies.

**Table 1:** Summary and comparative analysis of related studies.

Author	Method	Dataset type	Imaging modality	Input dim.	Best reported result
Ronneberger et al. (12)	Encoder–decoder U- shaped architecture with skip connections, elastic data augmentation, and a weighted loss function designed to better separate touching or adjacent objects.	Public dataset	EM, light microscopy	2D	Average IoU of 92.0% on PhC-U373 and 77.5% on DIC-HeLa.
Chen et al. (17)	A CNN–Transformer hybrid U-Net with patch-based Transformer encoding and query-based mask classification decoding, enhanced by cross-attention with CNN features and iterative coarse-to-fine mask refinement.	Public dataset	CT, MRI	2D/3D	Dice improvement of 1.06% (multi-organ segmentation) and 4.30% (pancreatic tumor segmentation) over nnU-Net.
Sun et al. (18)	A CNN–Transformer U- Net with Dual Attention Blocks that jointly model spatial and channel dependencies, applied in both encoder and skip connections to enhance feature refinement and reduce redundancy.	Public dataset	CT, X-ray, Dermoscopy, Endoscopy	2D/3D	Achieved a DSC of 79.80% and an HD of 23.48 mm on the Synapse dataset.
Bruschi et al. (19)	A Grad-CAM guided streamlined U-Net that concatenates GradCAM tumor activation heatmaps as an extra input channel and adopts Leaky ReLU, batch normalization and spatial dropout for lightweight feature encoding.	Public dataset	CT	2D	Achieved a Dice coefficient of 91.27% and an IoU of 86.26%.
Lan et al. (20)	A CNN–Transformer U-Net with Bi-level Routing Attention (BRA) for sparse global modeling, enhanced by BiFormer blocks and a channel–spatial attention skip connection module for efficient feature fusion and reconstruction.	Public dataset	CT, Dermoscopy, Endoscopy	2D	Achieved 82.47% DSC on Synapse.
Rezvani et al. (21)	A multi-scale fusion network that integrates channel-wise aggregation attention, multi-scale feature fusion, and refinement modules to enhance contextual feature representation and boundary refinement.	Public dataset	CT	2D	Achieved 98.04% IoU and 98.98% Dice on LungSegDBV1.
Fu et al. (22)	A CNN–Transformer U-Net with dual-path multi-scale feature extraction, lightweight channel-wise attention, and cross-stage feature fusion via MFF and cross-attention modules.	Public dataset	CT, Dermoscopy	2D/3D	Achieved 91.85% DSC on ISIC 2018, 86.21% avg DSC on Synapse, and 86.5% avg DSC on BTCV.
Hua et al. (23)	A 3D CNN U-Net with multi-kernel encoding, patch- merging downsampling, and enhanced feature modeling via multiangle skip connection interaction and global–local adaptive attention fusion.	Public and Private dataset	CT, MRI	3D	Achieved 87.77% DSC on ACDC, 81.65% DSC on BraTS19, and 87.64% DSC on temporal bone CT.
Guo et al. (24)	Res2Net-based UNet with dilated convolutions for multiscale feature extraction, ConvFormer for longrange dependency modeling, and channel attention-guided CFFB skip connections for improved feature fusion and segmentation accuracy.	Public dataset	CT	2D	Achieved 83.92% DSC and 14.51 mm HD on Synapse, and 93.27% DSC and 1.53 mm HD on Seg.A 2023.
Xu et al. (25)	Enhanced U-Net with collaborative channel–spatial attention, ASPP-based multi-scale feature fusion, asymmetric and dilated convolutions, and side-output deep supervision for robust medical image segmentation.	Public dataset	CT, MRI	2D	Achieved Dice scores of 72.64% (TCIA), 71.68% (MSD), 95.02% (KiTS19), and 79.32% (Cardiac).
Sun et al. (26)	Boundary DoU loss for boundary-aware medical image segmentation with adaptive size-dependent boundary weighting.	Public dataset	CT, MRI	2D	Synapse DSC 79.87% (Swin-UNet), ACDC DSC 91.29% (TransUNet), with improved boundary IoU (up to 87.78%/78.45%).
Lin et al. (27)	Dual-stream CNN–Transformer encoder with boundary guided decoder using Sobel operator for edge-aware segmentation refinement.	Public dataset	CT, Histopathology, Dermoscopy	2D	SOTA accuracy–efficiency trade-off across six medical image segmentation datasets.
Yang et al. (28)	Learns upper/lower dual boundary maps with complementary constraint and segmentation consistency supervision for structured retinal segmentation.	Private dataset	OCT	2D	Surpassing previous SOTA methods by 5.97% in average Dice and 10.83% in average BIoU.
Yu et al. (29)	The method uses gradient convolution (D- GConv and C-GConv) for boundary feature extraction, a GC branch for edge modeling, a U-shaped backbone for global features, BAM for channel attention, and a fusion module to combine global and local features.	Public dataset	Dermoscopy, Endoscopy, X-ray	2D	Improves Dice by up to 6.4% on ISIC2017 and IoU by 3.5% on ISIC2018; achieves Dice 0.909/0.898 (cup) and 0.987/0.983 (disc) on REFUGE/RIMONE.

## 3 Methods

### 3.1 Overall architecture

**Figure 1:**
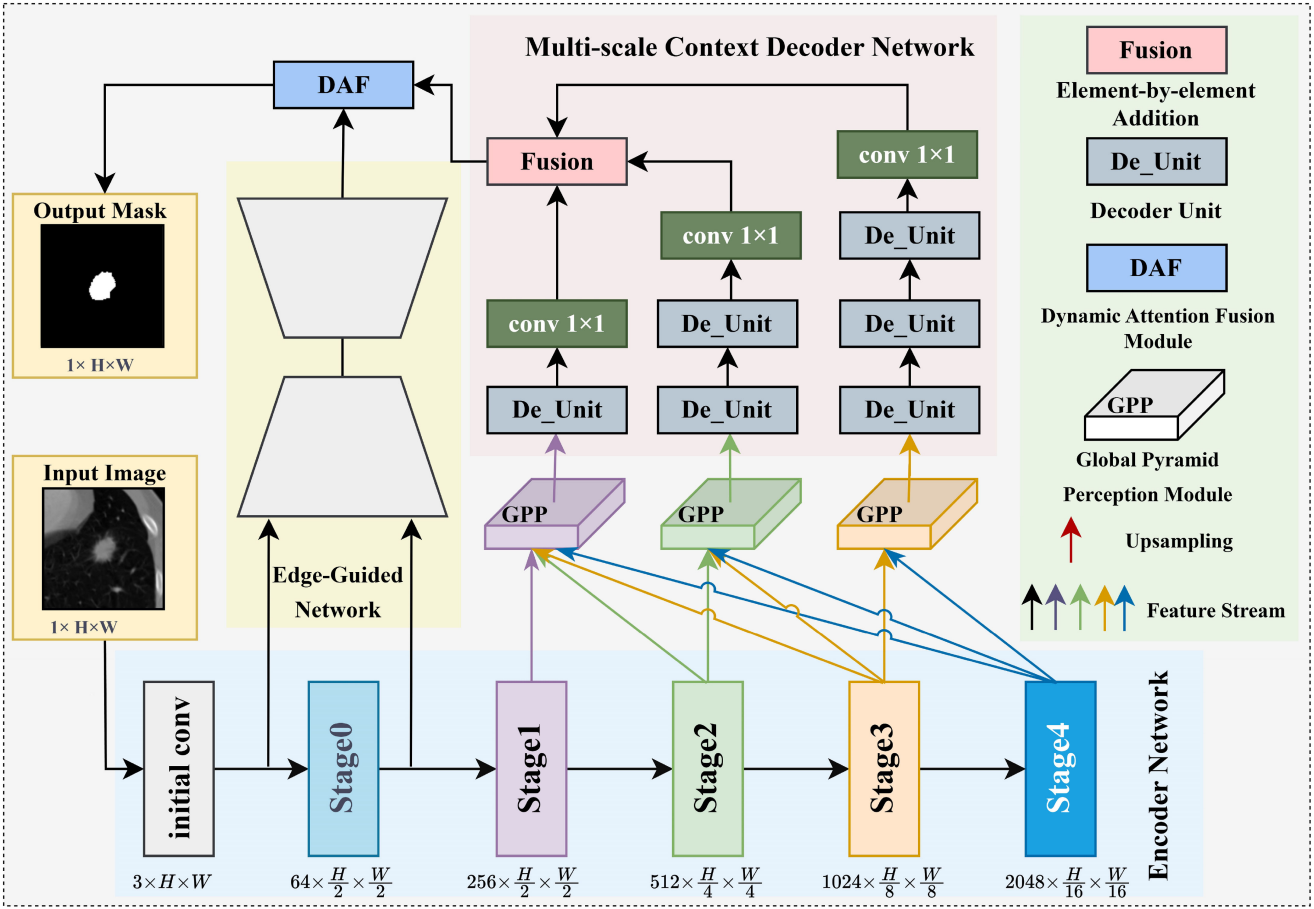
Here is a demonstration of the EGP-Net for lung nodule segmentation architecture, which consists of five key components: the encoder network, edge-guided network, global pyramid perception module, dynamic attention fusion module, and multi-scale context decoder network.

**Figure 1** illustrates the proposed Edge-Guided Pyramid Multi-Scale Contextual Attention (EGPNet) framework for lung nodule segmentation, comprising five key components: the encoder network, edge-guided network, global pyramid perception module(GPP), dynamic attention fusion module(DAF), and multi-scale context decoder network. The proposed EGP-Net is a 2D segmentation framework that operates on individual axial CT slices. Specifically, the 1 × 1 convolution first preprocesses the grayscale CT image of the lung nodule, yielding a feature denoted as ${\text{E}}_{1\times 1}$. This feature is fed into both the encoder and the edge-guided networks. Within the encoder network (using Res2Net-50 Gao et al. (30) as the backbone), multi-layer feature extraction is performed, yielding features denoted as $S_{i}\;(i=0,1,\ldots ,4)$. Notably, to better preserve the spatial structural information and coordinate changes in different dimensions, the initial pooling layer was skipped in the *S*_0_ operation, resulting in a feature resolution of $\frac{H}{2}\times \frac{W}{2}$ for the first layer. When *i >* 0, the feature resolution of the *i*-th layer was $\frac{H}{2^{i}}\times \frac{W}{2^{i}}$. To enhance the accurate capture of target boundary features, the framework incorporates an edge-guided network specifically designed for edge feature extraction. This network architecture first processes the semantic featuresE_1×1_ captured by 1 × 1 convolutions through a 3 × 3 convolutional kernel with stride 2. These features are then fused with low-level features *S*_0_ from the encoder network via max pooling. Subsequently, two identical sets of 3 × 3 convolutional blocks progressively extract the edge information and restore it to its original spatial resolution. Meanwhile, the multi-scale features extracted by the encoder were integrated into the GPP module. This stage combines feature maps with those from all higher-level stages to reconstruct skip connections, thereby promoting effective capture of global semantic information. The multi-scale contextual decoder integrates a Decoder Unit(De Unit) based on the Efficient Sub-Pixel Convolutional Neural Network to achieve super-resolution reconstruction. Finally, the local edge features captured by the edge-guided network and the global semantic features extracted by the multi-scale context decoder undergo adaptive weighted fusion through the DAF module to yield the final segmentation result. Each key step and its implementation are described below.

Furthermore, the computational efficiency of the proposed method is evaluated using model size and floating-point operations (FLOPs). FLOPs are measured based on a single forward pass with an input size of 128×128 pixels. The model size (MB) is calculated from the total number of learnable parameters assuming 32-bit floating-point precision. All competing approaches are tested under identical experimental environments and input settings for a fair comparison, and detailed analysis are listed in Section 5.2.

### 3.2 Global pyramid perception module

**Figure 2:**
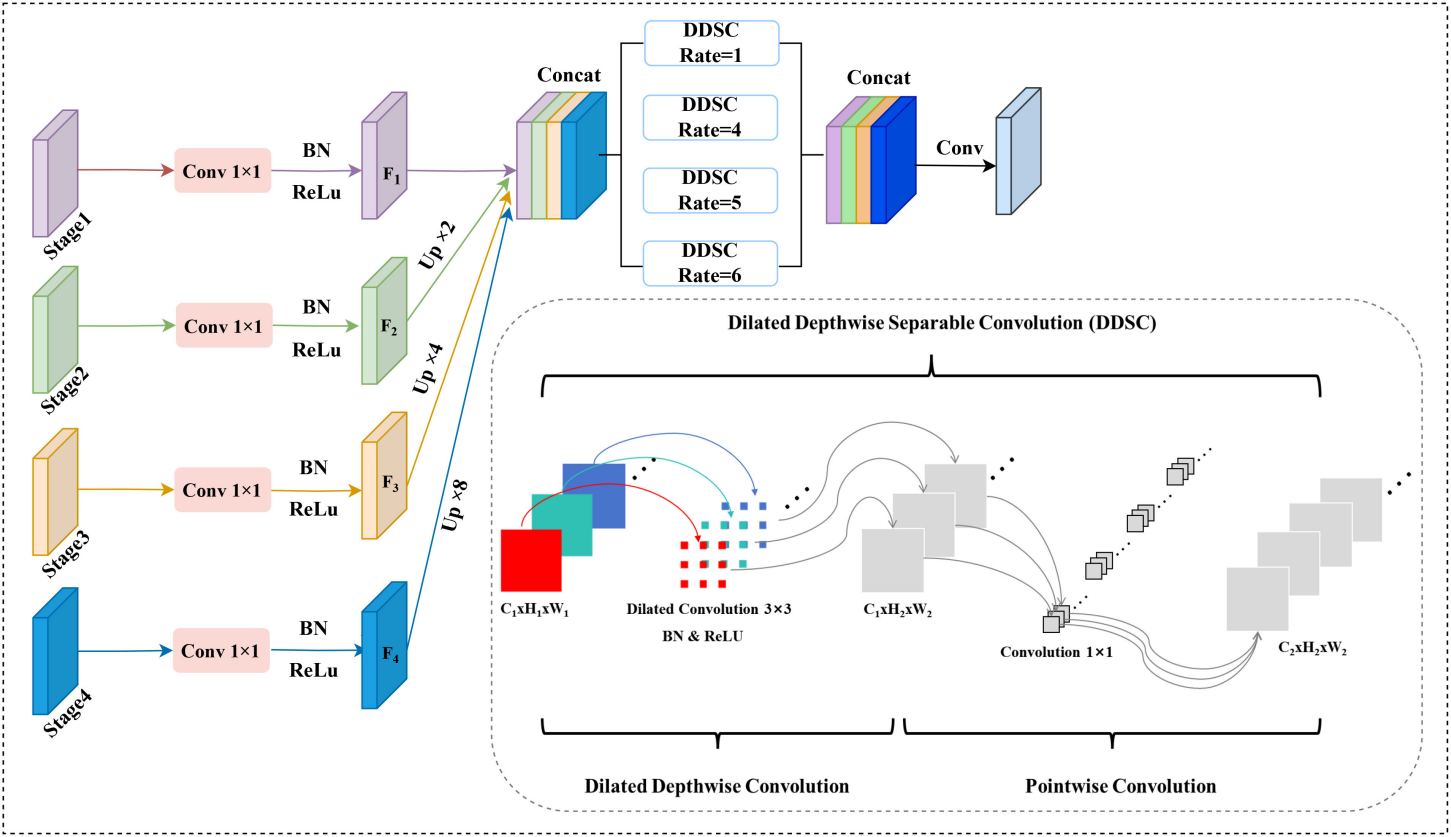
Schematic of the Global Pyramid Perception Module (GPP). Taking the skip connection reconstructed at stage 1 as an example, global information flows are transmitted to the multi-scale context decoder by fusing global contextual information from higher stages (stages 2, 3, and 4).

The encoder can learn global contextual information from input CT images, including the surrounding environment and category features of the lesions Zhu et al. (31). However, as this information propagates to shallower layers, it may gradually degrade Liu et al. (32). Furthermore, simple jump connections introduce certain interference factors and create semantic gaps owing to the mismatched receptive fields. To address these issues, we designed a Global Pyramid perception (GPP) module, as shown in **Figure 2**.

In the GPP module, the feature maps from the current stage are fused with those from all other higher-level stages to reconstruct the skip connection. Taking the GPP module in Stage 1 as an example, **Figure 2** illustrates the processing details. First, the feature maps from all stages are adjusted to the same channel space as Stage 1 using conventional 1×1 convolutions. Subsequently, the resulting feature maps F2, F3, and F4 are upsampled to the same spatial resolution as F1 and concatenated. To extract global features from feature maps at different stages, four improved dilated depthwise separable convolutions (DDSC) with different dilation rates (1, 4, 5, and 6) are applied in parallel. Notably, the number of stage branches is closely related to the expansion rate. Finally, the processed feature map is obtained through standard convolution operations. Similarly, the processing modules in the other stages can be summarized by Equation 1.

(1) $$G_{k}=\text{Conv}({\text{C}}_{j=1}^{m}({\text{DDSC}}_{r_{j}}({\text{C}}_{i=k}^{N}(\text{U}(\phi (F_{i}))))))$$

where *F_i_* denotes the feature map extracted from the *i*-th stage of the backbone network. *ϕ*(·) represents a channel alignment function implemented by a 1 × 1 convolution. U(·) denotes the bilinear upsampling operation, which is used to resize feature maps from different stages to the same spatial resolution as the current stage and degenerates to an identity mapping when *i* = *k*. C(·) denotes the concatenation operation along the channel dimension. DDSC*_rj_*(·) denotes the dilated depthwise separable convolution with dilation rate *r_j_*.

### 3.3 Dynamic attention fusion module

**Figure 3:**
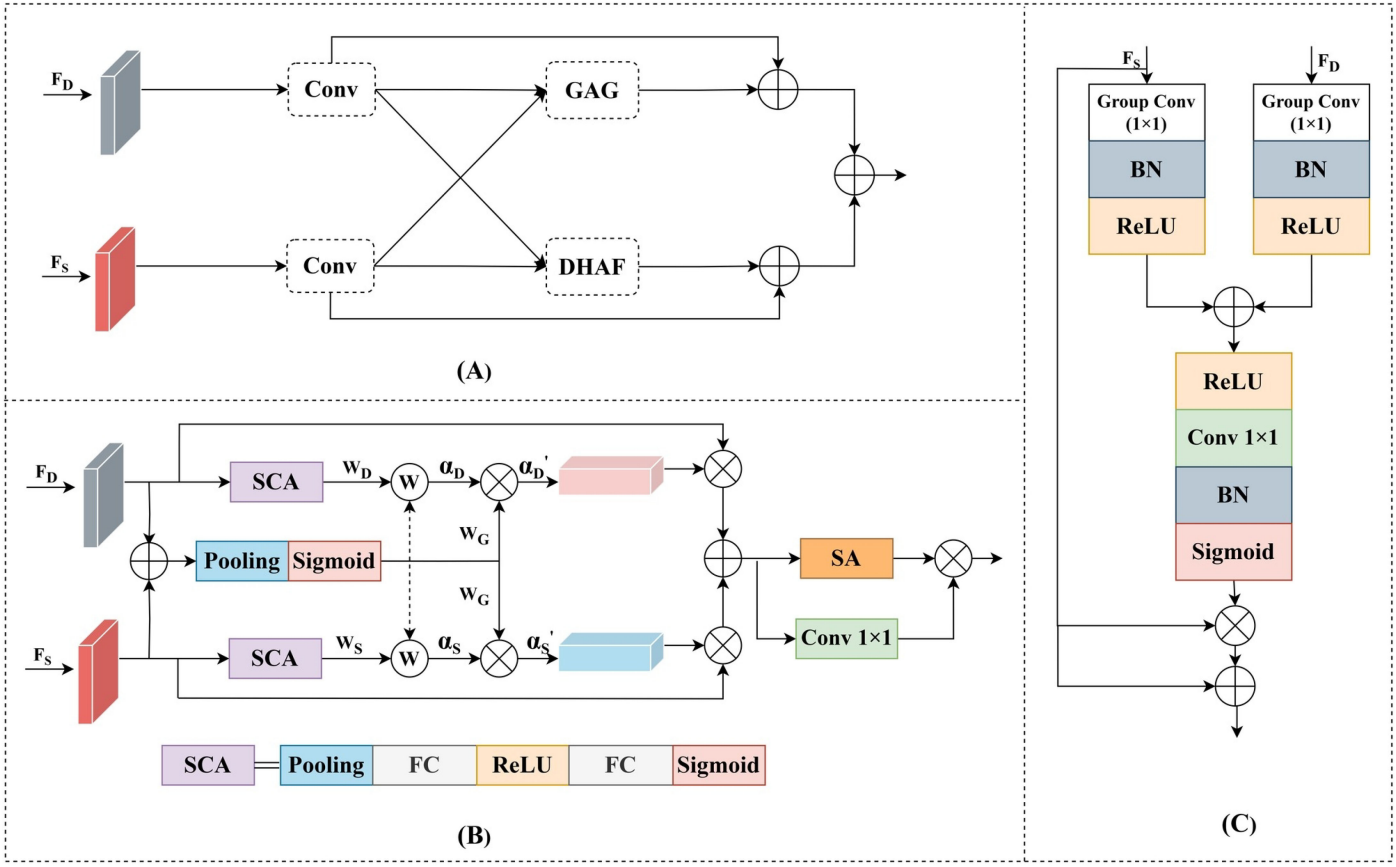
Structure of the DAF module. **(A)** Dynamic attention fusion (DAF). **(B)** Dual-branch hybrid attention fusion (DHAF). **(C)** Grouped attention gate (GAG).

Simply linearly combining features from different semantic levels through operations like (e.g., such as summation or concatenation) is often suboptimal. Thus, this study develops a dynamic attention fusion module (DAF) to capture the complex complementary relationships and semantic differences between global semantics and local edge features, fully preserving fine-grained edge information while effectively enhancing the global context, thereby improving the model’s overall segmentation performance. The architecture of the DAF and its internal components are illustrated in **Figure 3A**.

The first branch consists of a standard convolution and a grouped attention gate (GAG) module. The GAG module is designed to guide local features to focus on more discriminative regions in the spatial domain. The structure of the GAG module is shown in **Figure 3C**. Specifically, grouped 1 × 1 convolutions are applied to the guiding features and local features, respectively, to project them into a reduced intermediate feature space. The transformed features are then combined via element-wise addition followed by a nonlinear activation to generate a spatial attention mask. This mask is normalized using a Sigmoid function and subsequently applied to the local features, enabling adaptive enhancement of critical edge and structural information. In addition, a residual connection is introduced to prevent excessive suppression of important fine-grained details. The implementation of this module is given in Equations 2–4.

(2) $$F_{sɡ}=\sigma (\text{BN}(\text{GroupConv}(F_{s})))$$

(3) $$F_{Dɡ}=\sigma (\text{BN}(\text{GroupConv}(F_{D})))$$

(4) $$F_{\text{GAG}}=F_{s}\times (\delta ({\text{Conv}}_{1\times 1}(\sigma (F_{sg}+F_{Dg}))))+F_{s}$$

where *F_s_* and *F_D_* denote the input features, while BN(·) and *σ*(·) represent the batch normalization operation and the activation function, respectively.

The second branch is constructed with a standard convolution and a dual-branch hybrid attention fusion (DHAF) module. The structure of the DHAF module is shown in **Figure 3B**. Specifically, DHAF first introduces a lightweight channel attention mechanism to model the channel-wise importance of the two input feature streams independently. Global average pooling is employed to extract global contextual descriptors, which are then transformed through fully connected layers to generate corresponding channel attention weights. Based on these responses, the module dynamically computes adaptive fusion coefficients to balance the contributions of the two feature branches, enabling content-aware feature aggregation. After channel-wise weighted fusion, a spatial attention module is further incorporated to refine the fused features in the spatial domain, highlighting informative regions while suppressing irrelevant responses. The implementation of the formula is given in Equations 5–7.

(5) $$w_{D}=\text{SCA}(F_{D})$$

(6) $$w_{S}=\text{SCA}(F_{s})$$

(7) $$\text{SCA}=\delta (L(\sigma (L(\text{Pooling}(F_{i})))))$$

where SCA denotes the lightweight channel attention mechanism. *L*(·), *σ*(·), and *δ*(·) represent the fully connected layer, the activation function, and the Sigmoid operation, respectively.

Based on the feature maps produced by the SCA, the weighting coefficients *α_D_*and *α_S_*are computed. The calculation method is given in Equations 8, 9. where *θ* is a very small constant used to avoid division-by-zero errors. In addition, to achieve dynamic and content-aware feature fusion, a global attention weight *w_G_* is obtained through global pooling followed by a Sigmoid operation. The calculation formula for *w_G_* is given in Equations 10–13 define the calculation formula for the final feature fusion result.

(8) $$\alpha_{D}=\frac{w_{D}}{w_{D}+w_{S}+\theta }$$

(9) $$\alpha_{S}=\frac{w_{S}}{w_{D}+w_{S}+\theta }$$

(10) $$w_{G}=\delta (\text{pooling}(F_{s}+F_{D}))$$

(11) $${\alpha^{\prime }}_{D}=\alpha_{D}\times W_{G}$$

(12) $${\alpha^{\prime }}_{S}=\alpha_{S}\times W_{G}$$

(13) $$X={\alpha^{\prime }}_{D}\times F_{D}+{\alpha^{\prime }}_{S}\times F_{S}$$

Finally, this module incorporates a spatial attention(SA) mechanism to further enhance spatial hierarchical information. This mechanism applies weighted processing to spatial regions, guiding the network to focus on key areas while suppressing background information. The final output of the DHAF module is given in Equation 14.

(14) $$F_{\text{DHAF}}=\text{SA}(X)\times \text{Conv}(X)$$

### 3.4 Edge-guided network

**Figure 4:**
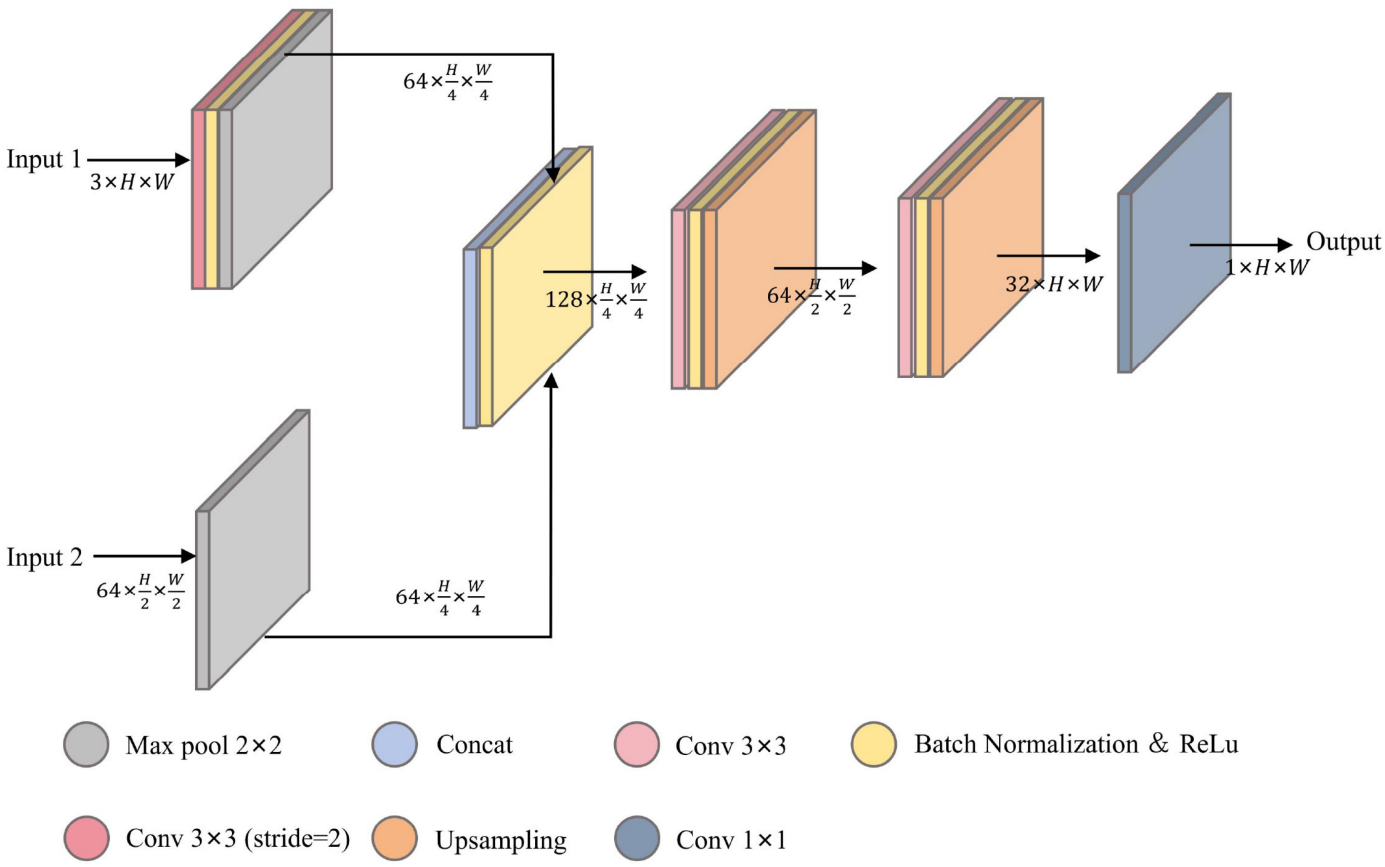
Structure of the edge-guided network. Shallow encoder features are effectively utilized to capture abundant boundary information.

Owing to their small size, lung nodules exhibit blurred boundaries in CT images and often visually overlap with surrounding structures such as blood vessels and bronchi, which can significantly impact segmentation results. To enhance segmentation accuracy, effectively extracting edge information is crucial. Therefore, this paper proposes an edge-guided network designed to extract and enhance local edge features. This aids the model in accurately reconstructing nodule contours at multiple scales while suppressing interference from surrounding structures, thereby improving segmentation boundary precision and robustness. The overall structure of the system is shown in **Figure 4**.

In the network design, lower-level features retain richer boundary information; therefore, this study fully utilizes information from the lower feature layers of the encoder. Specifically, the semantic features obtained through initial channel embedding via 1 × 1 convolutions are denoted as ${\text{E}}_{1\times 1}\in {\text{R}}^{H\times W\times 3}$. The low-level feature output of encoder stage 0 was ${\text{S}}_{0}\in {\text{R}}^{\frac{H}{2}\;\times \frac{W}{2}\;\times 64}$. Here, $E_{1\times 1}$ was first processed through a 3 × 3 convolution with stride 2 and 64 channels to reduce the feature map resolution to half its original size. Subsequently, batch normalization (BN), ReLU activation, and 2 × 2 max pooling were applied sequentially, yielding a feature map of size $\frac{H}{4}\;\times \;\frac{W}{4}\;\times \;64$. Concurrently, max pooling was performed on S_0_, producing a feature map $\frac{H}{4}\;\times \;\frac{W}{4}\;\times \;64$. These two outputs were concatenated along the channel dimensions to form the fused feature ${\text{F}}_{b1}\in {\text{R}}^{\frac{H}{4}\;\times \;\frac{W}{4}\;\times 128}$. Subsequently, F*_b_*_1_ was subjected to two identical sets of convolutional blocks. Each set comprised a 3 × 3 convolution, BN, ReLU activation, and bilinear interpolation with a 2×2 scale factor, which enhanced the resolution while halving the number of channels. The outputs of these convolutional blocks are denoted as F*_b_*_2_ and F*_b_*_3_, respectively. Finally, F*_b_*_3_ passes through a 1 × 1 convolutional layer to generate a single-channel boundary feature map ${\text{F}}_{\text{out}}\in {\text{R}}^{H\;\times W\times \;1}$.

### 3.5 Multi-scale context decoder network

The multi-scale context decoder network performs progressive decoding by integrating global context information guided by the GPP module. The GPP module reconstructs jump connections by integrating multi-level global context information, effectively mitigating semantic redundancy that might arise from simple concatenation. Jump connections at each stage originate from both the stage and the higher stages, facilitating the effective fusion of information across different stages. This approach was inspired by the skip-connection mechanism in U-Net, which effectively integrates low-level spatial information with deep semantic information. It ensures the deep fusion of low-to-high-level features and convergence of edge details, thereby enhancing the multi-scale feature extraction and detailed recovery capabilities of the network. This also provides a foundation for feature reconstruction during the subsequent spatial resolution recovery at different scales.

**Figure 5:**
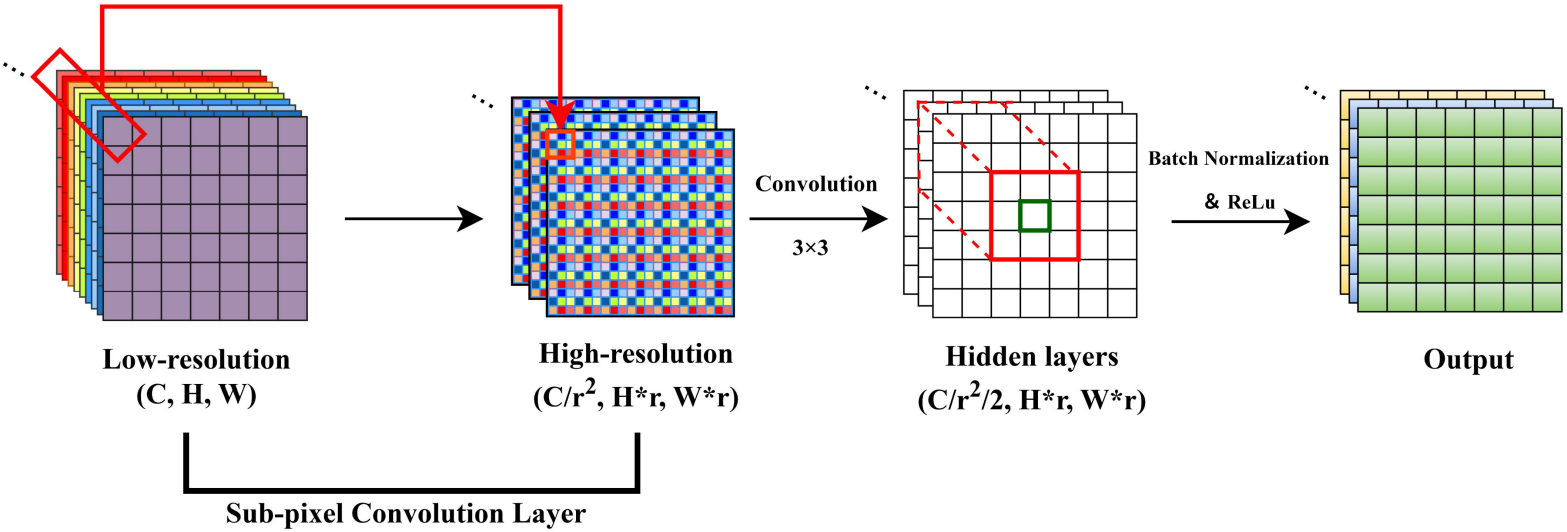
Basic de unit structure of the multi-scale context decoder network.

A fundamental Decoder Unit(De Unit) crucial to the multi-scale context decoder network was designed to extract high-level semantic information more efficiently and generate global features. This unit efficiently restores the image spatial resolution and generates global features. Specifically, **Figure 5** illustrates the structure of basic De Unit, where three branches guided by the GPP module employ an Efficient Sub-Pixel Convolutional Neural Network(ESPCN) for feature reconstruction. This architecture first uses a sub-pixel convolution layer to up-sample, generating feature maps of size (C*/*r^2^, r*H, r*W). Subsequently, a 3×3 convolution halves the number of feature map channels to extract more discriminative features. Finally, batch normalization and ReLU nonlinear activation normalize and activate the features to optimize the feature performance and enhance the model stability, thereby achieving high-quality output.

The ESPCN employed in this study reduces the blurring potential associated with traditional interpolation methods through efficient feature upscaling. It also achieves high computational efficiency and accuracy in high-resolution image reconstruction, enabling each branch to efficiently restore and enhance image details, thereby improving the overall modeling performance. In the experiments in this study, the subpixel convolution layer was implemented in PyTorch using the PixelShuffle function, with the parameter r set to 2. Where, r denotes the number of subpixels corresponding to each pixel in the subpixel convolution layer.

### 3.6 Loss functions

For the binary classification task in medical image segmentation, the fusion model employs both the Dice Similarity Coefficient (DSC) loss Litjens et al. (33) and the Binary Cross-Entropy (BCE) loss function. Owing to the significantly lower number of pixels occupied by nodules compared to the background in CT scans, the data exhibited a severe positive-negative sample imbalance. To address this challenge, we selected the Dice loss as the network evaluation metric, utilizing the intersection-over-union ratio between predictions and ground truth to quantify segmentation quality; a greater overlap indicates a lower loss. Compared with the traditional loss function, Dice loss is more suitable for severely imbalanced datasets because it prioritizes foreground region detection and effectively addresses extreme imbalances. However, when training on small object images, the Dice loss exhibits poor stability on small targets. The BCE loss function is commonly used for segmentation tasks. The formulae for Dice and BCE are shown in Equations 15, 16.

(15) $$L_{\text{Dice}}=1-\frac{2|X\cap Y|}{\left|X|+|Y\right|}$$

(16) $$L_{\text{BCE}}=-\frac{1}{N}\sum_{n=1}^{N}[w_{n}(y_{n}\text{log }x_{n}+(1-y_{n})\text{log }(1-x_{n}))]$$

where |X| and |Y| denote the sum of the positive pixels in the model’s output binary image and ground truth binary image, respectively. |X∩Y| represents the intersection of X and Y—the number of pixels that are positive in both the model output and ground truth. Coefficient 2 was used to prevent double counting. W is a hyperparameter, and y takes values of 0 or 1.

The loss function combines BCE loss and Dice loss, as shown in Equation 17.

(17) $$L={\text{λ}}_{1}\cdot L_{\text{BCE}}+{\text{λ}}_{2}\cdot L_{\text{Dice}}$$

## 4 Experiments

### 4.1 Datasets

Experiments were conducted using the publicly available LIDC and private MID-FAHGMU datasets to validate the effectiveness of the proposed lung nodule segmentation model.

The LIDC, compiled by the U.S. National Cancer Institute, includes 1,018 lung CT scans, each independently labeled and diagnosed by a panel of four senior radiologists, totaling 15,096 images. Detailed information regarding the training and testing data sources, along with annotation methods, is accessible via the link at Cancer Imaging Archive. When training models using the LIDC dataset, this study adopted the professional opinion of each radiologist to ensure fairness and validity of the experimental data. By performing logical OR operations on the regions labeled as nodules, a comprehensive nodule mask image is generated to enhance the accuracy of the nodule region.

The MID-FAHGMU private dataset comprises 1,610 lung CT slices provided by the Department of Medical Imaging at Liuzhou Workers’ Hospital. This dataset includes 404 solid nodules, 344 partially solid nodules, 457 calcified nodules, and 405 ground-glass nodules. These CT images had a resolution of 512 × 512 pixels and were acquired following standard imaging protocols using multi-slice spiral CT scanners. To ensure data diversity, the dataset originates from different patient cohorts and scanning devices, introducing variability (e.g., scanning angles, noise levels, and image quality) that increases segmentation challenges. During data processing, raw DICOM files were first transformed into PNG images through the MicroDicom software. Subsequently, pixel-level mask annotations of lung nodules were performed by radiologists using LabelMe. All cases were independently annotated by three radiologists with more than 10 years of experience in thoracic CT interpretation and subsequently reviewed by a senior radiologist. To evaluate annotation consistency, an inter-observer agreement analysis was conducted, and the Dice Similarity Coefficient (DSC) was used for quantitative assessment. The results indicated a high level of agreement among the observers. Annotation discrepancies were first resolved through consensus discussion; cases that remained controversial after discussion were adjudicated by the senior radiologist, whereas samples for which a consensus could not be reached were excluded. Multiple rounds of review and validation were conducted throughout the annotation process to ensure annotation quality and consistency.

**Figure 6:**
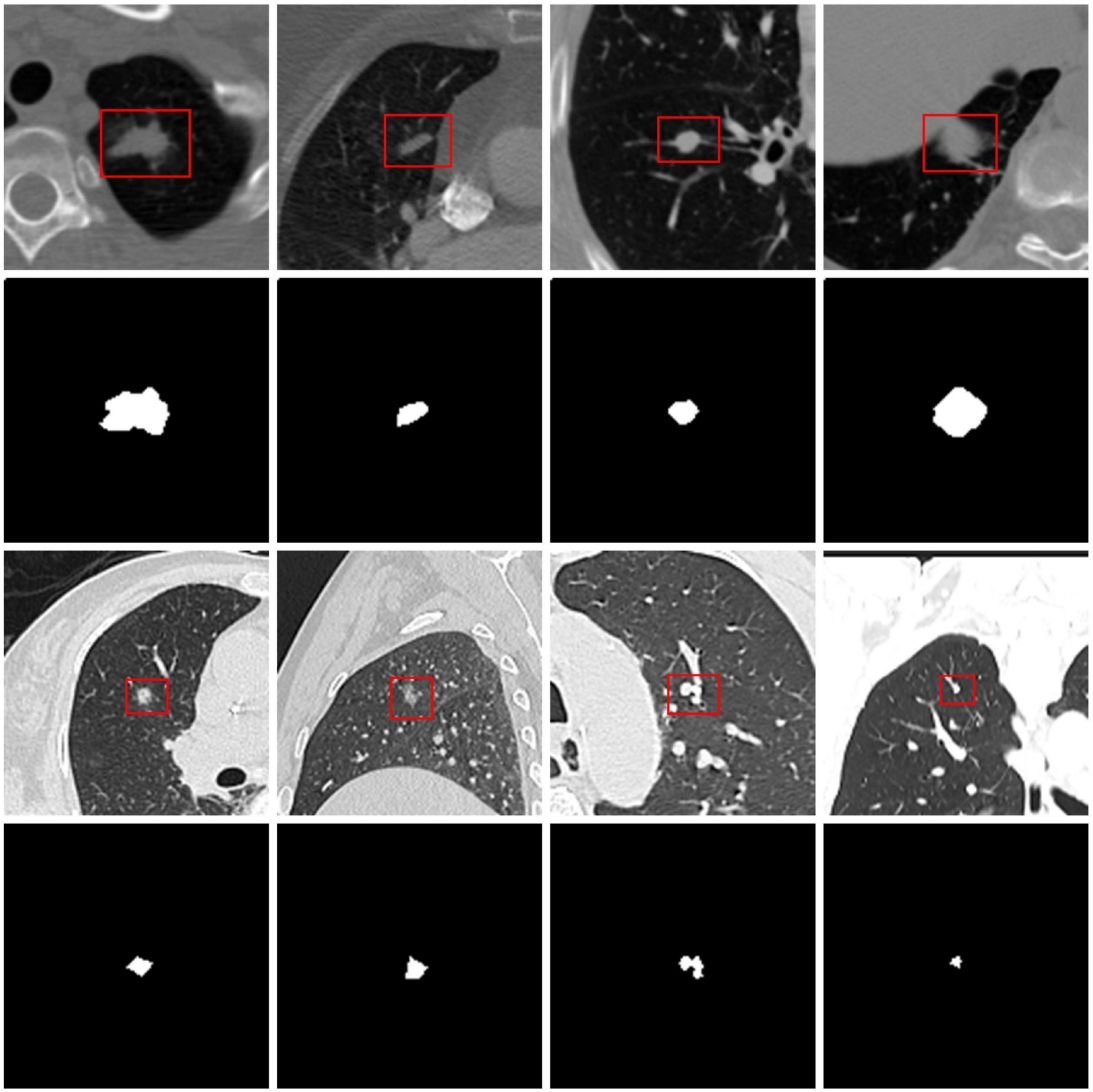
Shows the lung nodule datasets used in the experiments: the upper half is the LIDC public dataset, and the lower half is the MID-FAHGMU private dataset.

In the experiment, 15,096 preprocessed lung nodule CT images from the LIDC public dataset were randomly split at a 6:2:2 ratio for training (9,057 images, 60%), validation (3,019 images, 20%), and testing (3,020 images, 20%). Similarly, 1,610 lung nodule CT images from the MID-FAHGMU private dataset were randomly split in a 6:2:2 ratio for training (966 images, 60%), validation (322 images, 20%), and testing (322 images, 20%). Notably, to prevent data leakage, the dataset partitioning is strictly performed at the patient level, ensuring that CT images or nodules from the same patient are not assigned to different subsets. The optimal model parameters were saved based on the validation set results and were evaluated using the test set. **Figure 6** shows examples from both datasets. **Table 2** summarizes detailed characteristics of the used datasets.

**Table 2:** Summarizes detailed clinical and imaging characteristics of the used datasets.

Characteristic	LIDC-IDRI	MID-FAHGMU
Age	N/A	49.2 ± 14.8
Sex	N/A	Male (58.4%), Female (41.6%)
Histology	N/A	Adenocarcinoma (56.2%), Squamous cell carcinoma (24.3%), Benign (19.5%)
Slice thickness	0.6mm–5.0mm	1.0mm–3.0mm
Slice spacing	0.6mm–3.0mm	1.0mm–2.0mm
CT acquisition	Multi-center helical/spiral thoracic CT	Multi-slice spiral thoracic CT

#### 4.1.1 Data preprocessing

Before model training, all two-dimensional CT images underwent a unified preprocessing pipeline. First, image patches were cropped with the nodule as the center and resized to a fixed resolution of 128 × 128 pixels along the slice dimension only. Each slice was independently fed into the network, and no standardization of inter-slice physical spacing was performed. Subsequently, bias field correction was applied to reduce low-frequency intensity inhomogeneity caused by variations in scanning devices and imaging conditions. Then, bilateral filtering was used for noise suppression. After that, intensity normalization with a mean of 0.2 and a standard deviation of 0.15 was applied, mapping pixel values to the range [0, 1]. For the MID-FAHGMU dataset, during the conversion of raw DICOM data into PNG images, the MicroDicom software was used to uniformly apply lung window settings (window width WW = 1500 HU, window level WL = -600 HU) to enhance the visibility of pulmonary nodules and surrounding structures.

#### 4.1.2 Data augmentation

To mitigate overfitting and improve model generalization, data augmentation was performed to simulate various conditions encountered in clinical practice. Since the input to the network consists of nodule-centered cropped image patches rather than full CT slices, augmentation was applied at the patch level to better capture local morphological variations of pulmonary nodules while reducing interference from irrelevant background information. Specifically, horizontal flipping was applied with a probability of p = 0.5. In addition, random brightness and contrast adjustments were performed with a probability of p = 0.3 within a range of ±0.10. Gaussian noise with zero mean and variance in the range of [5, 25] was added with a probability of p = 0.2. Importantly, all augmentation operations were applied synchronously to both the original images and the corresponding ground-truth masks to ensure strict spatial alignment between images and labels.

### 4.2 Experimental details

All experimental codes in this study were implemented using Python 3.8 with PyTorch 2.4.1 and CUDA 11.8. Model training was conducted on a Windows 10 laptop equipped with a 12th Gen Intel(R) Core(TM) i5-12400F CPU and an NVIDIA GeForce RTX 3060 GPU. The method proposed in this paper is trained using Res2Net-50 pre-trained weights. For the LIDC public dataset, the model was trained for 100 epochs with a batch size of 8. The initial learning rate was set to 5 × 10^−5^, and a cosine annealing strategy was employed to gradually decrease the learning rate to 5 × 10^−6^. The Adam optimizer was adopted with a weight decay of 1 × 10^−4^. To ensure reproducibility, the random seed was fixed at 2023 for all experiments. The training hyperparameters on the MID-FAHGMU private dataset were consistent with those used for the LIDC dataset. It is worth noting that the hyperparameter settings in this study were adopted from our previous work Lu et al. (34), where the relevant parameters were determined and validated through extensive experimental studies. No additional hyperparameter tuning was performed in this study.

### 4.3 Evaluation metrics

In this study, we employed IoU, the Dice coefficient, and the F*_β_*-score (*β* ∈ {0.5,2}) as evaluation metrics for a comprehensive experimental comparison. Specifically, the four evaluation metrics are defined as follows:

The Intersection over Union(IoU) is a commonly used metric for evaluating the performance of the model. It assesses segmentation accuracy by computing the intersection divided by the union of the predicted and ground truth masks, reflecting how closely the prediction matches the actual result. The overall IoU score for the model was obtained by averaging the IoU values across all categories. The formula for the IoU is expressed as follows (Equation 18).

(18) $$\text{IoU }=\;\frac{1}{n}\sum_{\text{i}=1}^{n}\frac{TP_{i}}{TP_{i}+FP_{i}+FN_{i}}$$

The Dice coefficient was used to evaluate the degree of agreement between the segmentation results and true labels, reflecting both overlap and consistency. The formula for the Dice coefficient is as follows (Equation 19).

(19) $$\text{Dice }=\;\frac{1}{n}\sum_{\text{i}=1}^{n}\frac{2\cdot TP_{i}}{2\cdot TP_{i}+FP_{i}+FN_{i}}$$

The F*_β_*-score combines precision and recall by adjusting the *β*-value to balance the importance of the two metrics. When *β >* 1 (e.g., *β* = 2), recall is prioritized, and when *β <* 1 (e.g., *β* = 0.5), precision is emphasized. The formula for the F*_β_*-score is expressed as follows (Equation 20).

(20) $$\begin{array}{ll} \text{precision} & =\frac{1}{n}\sum_{i=1}^{n}\frac{TP_{i}}{TP_{i}+FP_{i}},\text{ recall}=\frac{1}{n}\sum_{i=1}^{n}\frac{TP_{i}}{TP_{i}+FN_{i}}\\ F_{\beta } & =\frac{(1+\beta^{2})\cdot precision\cdot recall}{(\beta^{2}\cdot precision)+recall} \end{array}$$

In Equations 18–20, n denotes the total number of classes. TP*_i_*represents the area of the regions predicted as class i that overlap with the ground truth mask. FP*_i_*denotes the area of the regions predicted as class i that do not overlap with the ground truth mask. FN*_i_*indicates the area of regions not predicted as class i but should have been predicted as class i.

### 4.4 Ablation studies

#### 4.4.1 Ablation study of the module

In this study, the performance of each model within the proposed network architecture was comprehensively evaluated using the LIDC public dataset. Specifically, certain modules within the network were progressively removed or replaced, and the performance after structural changes was compared to assess the impact of each component on overall performance. The baseline model is a basic lung nodule segmentation network without any enhanced modules. The model employs a pretrained Res2Net-50 as the encoder, while the decoder is composed of basic De Unit. In addition, standard skip connections and basic feature concatenation structures are retained, without introducing the edge-guided network, GPP module, or DAF module. These experimental results provide valuable insights for optimizing and improving network architecture. The effectiveness of each module is analyzed below.

#### 4.4.2 Effectiveness of GPP

In the experiments, to determine an appropriate configuration of the dilation rate parameters in the GPP, we designed and conducted multiple ablation studies with different dilation rate combinations (see **Table 3**). By systematically comparing the segmentation performance of the model under various dilation rate settings, the optimal parameter combination was selected. The experimental results indicate that the dilation rate configuration of 1, 4, 5, and 6 effectively enlarges the receptive field while preserving local detail information, thereby enhancing global context modeling capability and further improving the segmentation performance of the network. In contrast, other configurations, such as dilation rates set to 1, 2, 4, and 8, may introduce excessive irrelevant background noise, thereby weakening the model’s ability to focus on the target region.

**Table 3:** Ablation experiments on different dilation rate configurations in GPP based on the LIDC dataset, where ↑ indicates that higher metric values are better.

Different dilation rates	Evaluation index			
	IoU (%)↑	Dice (%)↑	F2-score (%)↑	F0.5-score (%)↑
(1 2 3 4)	87.86	92.35	92.67	92.80
(1 2 4 8)	88.13	92.51	92.79	93.02
(1 3 5 7)	87.93	92.33	92.62	92.72
(1 3 4 5)	88.15	92.43	92.77	92.95
**(1 4 5 6)**	**88.32**	**92.65**	**92.88**	**93.11**
(1 4 6 8)	88.19	92.48	92.76	93.03
(1 5 6 7)	88.05	92.55	92.80	93.06

The best-performing metrics are highlighted in bold.

**Figure 7:**
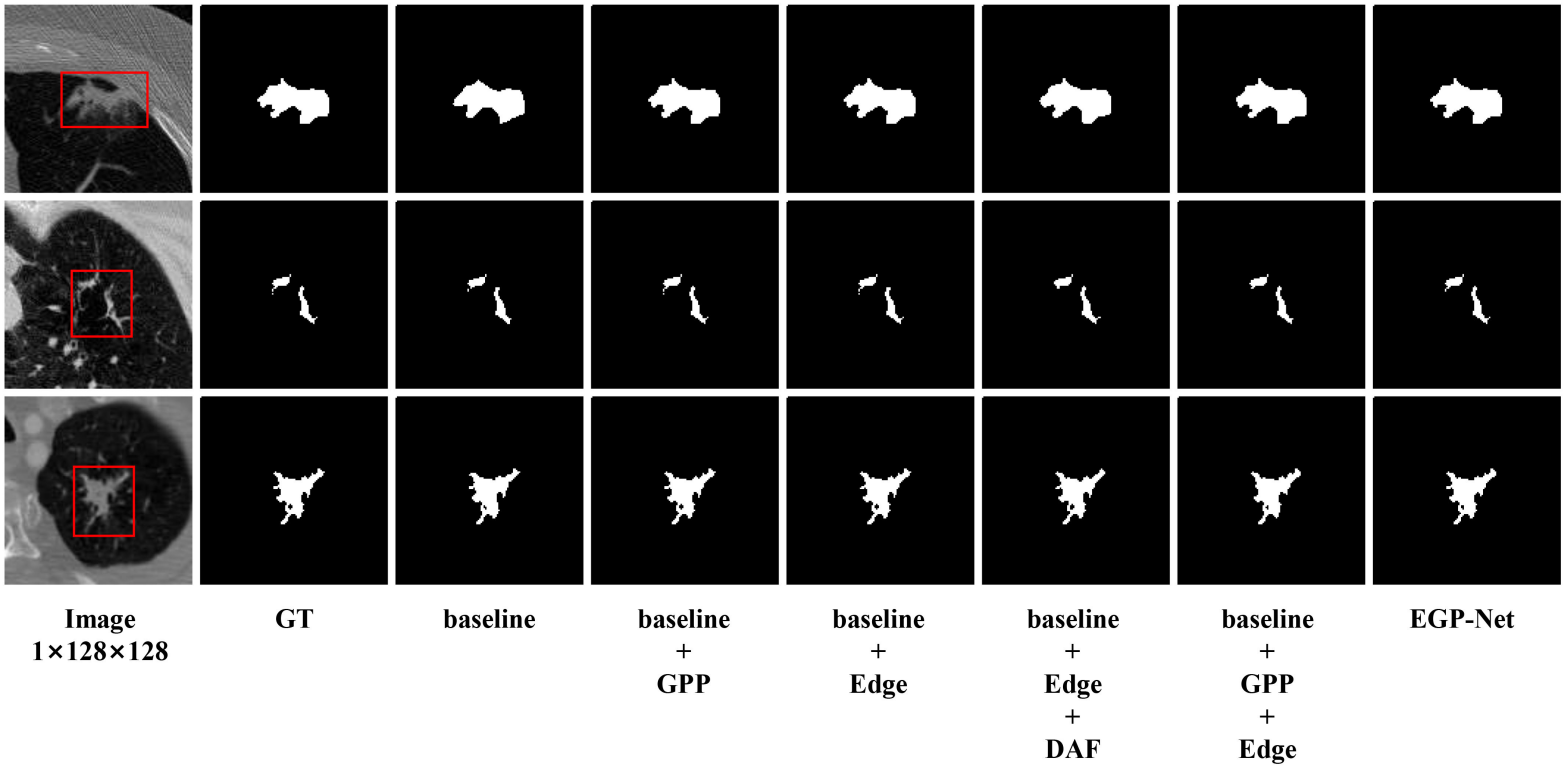
Visualization results of ablation experiments.

In the proposed network architecture, the GPP module aims to mitigate the semantic gap and irrelevant noise issues inherent to classical skip connections. This is achieved by constructing global guidance information through a multi-scale pyramid, systematically propagating high-level semantic information to lower layers. This approach maintains consistency across different feature scales and enhances the multi-scale context awareness. To investigate the effectiveness of the GPP module, we conducted ablation experiments, the results of which are presented in **Table 4**. This approach includes both isolated ablation of the GPP module and joint ablation with other components (e.g., edge guidance network and DAF module). The experimental results demonstrated that incorporating the GPP module significantly improved the lung nodule segmentation performance of the entire network framework. Visual comparisons in **Figure 7** further reveal that adding the GPP module optimizes the feature extraction process, generating more discriminative features and thereby enhancing the segmentation performance.

**Table 4:** Ablation experiments using the LIDC public dataset to evaluate the effectiveness of different key components, where ↑ indicates that higher metric values are better.

Model name	Modules			Evaluation metrics			
	GPP	Edge	DAF	IoU (%)↑	Dice (%)↑	F2-score (%)↑	F0.5-score (%)↑
baseline				86.53	91.68	91.89	92.37
baseline+GPP	✓			87.69	92.31	92.14	92.65
baseline+Edge		✓		87.64	92.24	92.45	92.63
Baseline+Edge+DAF		✓	✓	87.92	92.43	92.51	92.85
baseline+GPP+Edge	✓	✓		88.03	92.44	92.35	93.08
EGP-Net	✓	✓	✓	**88.32**	**92.65**	**92.88**	**93.11**

With the best performance metrics highlighted in bold for clarity and emphasis.

#### 4.4.3 Effectiveness of DAF

To verify the effectiveness of the DAF module in fusing multi-level semantic features from the multi-scale context decoder and the edge-guided network, ablation experiments were conducted on its core sub-modules, as reported in **Table 5**. It can be observed that, without introducing any attention-based fusion mechanism, the model exhibits relatively limited performance in terms of IoU and Dice, indicating that simple feature aggregation fails to fully exploit the complementary information across different semantic levels. When only the GAG sub-module is incorporated, noticeable improvements are achieved in IoU, and Dice, demonstrating that the grouped attention gate effectively facilitates the interaction between high-level semantic information and low-level edge details, thereby enhancing the model’s response to lung nodule regions. Similarly, introducing only the DHAF sub-module leads to consistent performance gains, suggesting that the dual-branch hybrid attention fusion mechanism benefits from dynamic weighting to emphasize discriminative regions while suppressing redundant interference. When both GAG and DHAF are jointly employed, the model attains the best performance across all evaluation metrics. These results indicate that the DAF module can effectively integrate multi-scale contextual information with edge-guided features, significantly strengthening local structural and boundary representations while preserving global semantic consistency, thereby improving overall lung nodule segmentation performance.

**Table 5:** Ablation experiment results on different components of DAF based on the LIDC dataset, where ↑ indicates that higher metric values are better.

Methods	GAG	DHAF	IoU (%)↑	Dice (%)↑	F2-score (%)↑	F0.5-score (%)↑
EGP-Net	×	×	88.05	92.43	92.37	92.99
	×	✓	88.18	92.54	92.67	93.06
	✓	×	88.22	92.60	92.78	93.10
	✓	✓	**88.32**	**92.65**	**92.88**	**93.11**

With the best-performance metrics highlighted in bold for clarity and emphasis.

Furthermore, to validate the effectiveness of the DAF module within the EGP-Net framework, additional ablation experiments were conducted on the LIDC dataset, with results summarized in **Table 4**. The results demonstrate that embedding the DAF module enables the overall network to more effectively integrate features from different semantic levels, leading to a notable improvement in lung nodule segmentation accuracy. The qualitative results presented in **Figure 7** further corroborate these findings, where clearer nodule boundaries and more accurately delineated fine protrusion structures are observed, resulting in a substantial enhancement in segmentation precision. These results collectively confirm that the proposed dynamic attention fusion module effectively enhances performance in lung nodule segmentation tasks.

#### 4.4.4 Effectiveness of the edge-guided network

Within the constructed lung nodule segmentation model, the edge-guided network focuses on extracting local edge information and enhancing the expressive power of low-level features. Injecting these highly responsive edge features into the attention-based feature fusion module, it compensates for performance losses caused by blurred boundaries in traditional semantic segmentation, thereby improving segmentation task performance. To evaluate the effectiveness of the edge-guided network comprehensively, ablation experiments were designed, and the results are listed in **Table 4**. The ablation experiments encompassed scenarios in which the edge-guided network was removed and combined with other key modules. The results demonstrate that incorporating the edge-guided network significantly improves segmentation performance. For instance, when combined with the DAF module, the segmentation performance surpasses that achieved using the edge-guided network alone. When paired with the GPP module, the segmentation performance exceeds that of the standalone edge-guided network but remains inferior to that of the integrated network framework. Thus, when the edge-guided network, GPP, and DAF modules work together, the overall performance reaches its optimal state, with all the evaluation metrics showing the best results. This demonstrates that synergistic effects and complementary advantages among the modules play a crucial role in improving the segmentation performance, further highlighting the importance of this module combination within the model architecture and providing robust support for accurate lung nodule segmentation. The visualization results in **Figure 7** clearly demonstrate the impact of combining different modules on the performance of the segmentation model. Notably, when multiple modules collaborate, the model achieves a finer delineation of nodule contours and details, highlighting its unique role in boundary localization and boundary restoration, thereby further enhancing segmentation performance.

#### 4.4.5 Loss function weight comparison

To evaluate the performance of the two loss functions under different weights, we designed five combinations of loss functions, including BCE-only, Dice-only, and mixed losses with different weight settings. The corresponding performance is shown in **Table 6**.

**Table 6:** Comparison of different loss functions.

Loss function	IoU (%)↑	Dice (%)↑	F2-score (%)↑	F0.5-score (%)↑
*L*BCE	87.24	90.17	91.99	92.23
*L*DICE	87.74	92.36	92.44	92.95
*L*BCE + *L*DICE	**88.34**	92.51	92.54	93.03
0.5*L*_BCE_ + *L*DICE	88.32	**92.65**	**92.88**	**93.11**
*L*_BCE_ + 0.5*L*DICE	88.11	92.43	92.46	93.01

With the best-performance metrics highlighted in bold for clarity and emphasis. Where ↑ represents the advantage of a higher metric.

Experimental results indicate that the combination of 0.5*BCE + Dice is the most suitable for this study, achieving an IoU of 88.32% and a Dice coefficient of 92.65%. This configuration leverages the Dice loss to capture edge details more accurately while maintaining the stability of BCE gradients, thereby ensuring overall segmentation stability. Overall, the reasonable combination of BCE and Dice loss not only mitigates the class imbalance problem but also significantly improves the segmentation accuracy of the foreground regions, thereby enhancing the overall performance of the model in segmentation tasks.

## 5 Discussion

### 5.1 Main findings

To comprehensively validate the performance of the proposed lung nodule segmentation network framework, this study conducted comparative experiments with ten advanced medical image segmentation methods. The comparison methods include U-Net Ronneberger et al. (12), Attention U-Net Schlemper et al. (35), UNet3+ Huang et al. (36), SAR-U-Net Wang et al. (37), SwinUnet Cao et al. (38), UCTransNet Wang et al. (15), M2SNet Zhao et al. (39), TransUNetChen et al. (17), DA-TransUNetSun et al. (18), and BRAU-Net++ Lan et al. (20). The source code for these networks has been publicly released in relevant literature, enabling unified training and testing in this study. To ensure a fair comparison, all methods use the same data splits, data augmentation pipeline, and hyperparameter settings.

It should be noted that qualitative evaluation was conducted by two radiologists, one of whom is a chief physician with rich clinical experience. Detailed analyses are provided in the following sections.

#### 5.1.1 Performance on the LIDC dataset

As illustrated in **Table 7**, the proposed EGP-Net is compared with ten representative state-of-the-art methods on the LIDC public dataset. The comparison employed four evaluation metrics: IoU, Dice, F2-score, and F0.5-score. The data clearly demonstrate that EGP-Net outperformed all other methods across all evaluation metrics, proving its superior performance in lung nodule segmentation. First, regarding IoU, EGP-Net achieved 88.32%, which is significantly higher than that of the other models. For instance, the best-performing method UNet3+ achieved an IoU of 87.68%, which is still lower than that of EGP-Net. This indicates the distinct advantage of EGP-Net in accurately segmenting target regions, enabling the superior identification and isolation of lung nodule areas. In contrast, other methods such as Attention U-Net and U-Net perform poorly on nodules with complex shapes or blurred boundaries, often resulting in inaccurate segmentation or missed detections. These methods typically fail to effectively identify nodule boundaries when images exhibit high noise or poor contrast between the nodules and surrounding tissue.

**Table 7:** Evaluation of different methods on LIDC public dataset, where ↑ indicates that higher metric values are better.

LIDC					
Method	Source	Evaluation metrics			
		IoU (%)↑	Dice (%)↑	F2-score (%)↑	F0.5-score (%)↑
U-Net Ronneberger et al. (12)	MICCAI	86.17	91.53	91.52	92.09
Attention U-Net Schlemper et al. (35)	MIA	84.86	90.72	90.74	91.49
UNet3+ Huang et al. (36)	ICASSP	87.68	92.33	92.62	92.77
SAR-U-net Wang et al. (37)	CMPB	85.52	91.02	91.21	91.71
Swin-Unet Cao et al. (38)	ECCV	73.70	83.37	83.96	84.34
UCTransNet Wang et al. (15)	AAAI	86.04	91.42	91.55	92.06
M2SNet Zhao et al. (39)	TMI	83.10	89.34	90.14	89.75
TransUNet Chen et al. (17)	MIA	85.43	90.87	90.98	91.64
DA-TransUNet Sun et al. (18)	FIBB	84.39	90.20	90.37	91.06
BRAU-Net++ Lan et al. (20)	IEEE	86.63	91.53	91.62	92.18
EGP-Net	Our	**88.32**	**92.65**	**92.88**	**93.11**

With the best-performance metrics highlighted in bold for clarity and emphasis.

Second, EGP-Net also excels in the Dice coefficient performance, achieving a score of 92.65%. This significantly surpasses other methods, particularly the Swin-Unet (83.37%) and M2SNet (89.34%) models. The advantage EGP-Net in this aspect is particularly pronounced. In contrast, Swin-Unet and M2SNet exhibited noticeable performance degradation when handling small nodules or overlapping adjacent structures. Swin-Unet’s limitations primarily lie in its weaker ability to capture small nodules and those with blurred boundaries, whereas M2SNet has limited segmentation performance for complex nodules, leading to reduced segmentation quality.

EGP-Net also achieved outstanding results on the F2-score, and F0.5-score metrics, at 92.88%, and 93.11%, respectively. These metrics reflect the model’s comprehensive performance in terms of precision and recall, with the F0.5-score placing greater weight on the precision. The outstanding performance of EGP-Net on this metric demonstrates that the model maintains good recall while ensuring high precision, making it suitable for clinical applications that require high sensitivity. In contrast, other methods exhibit a noticeable trade-off between high precision and recall. Many approaches struggle to balance both, particularly in clinical applications that demand high sensitivity, which often leads to false negatives or false positives.

**Figure 8:**
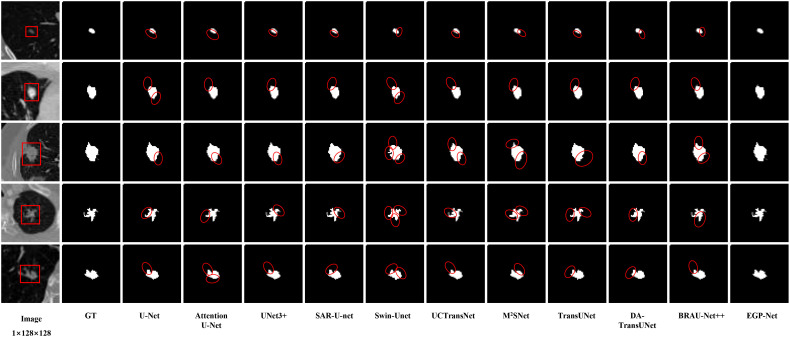
Visualization results of multiple methods on the LIDC dataset.

As shown in **Figure 8**, EGP-Net was qualitatively compared with ten representative segmentation methods on the LIDC dataset. Overall, EGP-Net produces segmentation results that are generally closer to the ground-truth (GT) annotations across a range of challenging cases. Compared with U-Net, EGP-Net preserves boundary details more effectively, particularly for large nodules. U-Net exhibits over- or under-segmentation in rows 2 and 3, while its segmentation in row 4 deviates noticeably from the GT. Attention U-Net shows reduced accuracy in row 2 and struggles with the complex nodules in rows 3 and 4. UNet3+ exhibits segmentation deviations in rows 1–3 and less accurate boundary delineation in row 5. SAR-U-Net shows boundary inaccuracies in row 2 and is affected by the complex background in row 3. Similar limitations can also be observed in Swin-Unet, UCTransNet, M2SNet, TransUNet, DA-TransUNet, and BRAU-Net++, particularly when handling complex structures and indistinct boundaries. By comparison, EGP-Net provides more accurate boundary delineation and better preserves the structural characteristics of nodules across these challenging scenarios. These visual results are consistent with the quantitative evaluation and further demonstrate the effectiveness of EGP-Net in segmenting nodules with complex morphology and blurred boundaries.

In addition to the aforementioned comparative experiments, as shown in **Table 8**, we also compared multiple state-of-the-art lung nodule segmentation algorithms on the lung nodule segmentation task with the LIDC dataset. The comparative results demonstrate that the proposed method exhibits superior performance in lung nodule segmentation. Specifically, REMU-Net Li et al. (40) achieves an average IoU of 86.25% and an average Dice of 84.76%. AC-UNet Zhang et al. (41) obtains an average IoU and Dice of 86.47% and 84.44%, respectively. MDFN Cai et al. (16) reaches an average IoU of 80.78% and an average Dice of 89.19%. FOC-Net Li et al. (42) achieves an average IoU of 85.37% and an average Dice of 92.06%. In contrast, the proposed method achieves an average IoU and Dice of 88.32% and 92.65%, respectively, outperforming these existing methods and further demonstrating its higher effectiveness in lung nodule segmentation tasks. All these comparison results are cited from the original literatures.

**Table 8:** Comparison experimental results under the same conditions.

Existing deep learning related work	Source	IoU (%)↑	Dice (%)↑
REMU-Net Li et al. (40)	Physical and Engineering Sciences in Medicine (2022)	86.25	84.76
AC-UNet Zhang et al. (41)	Proc. 5th Int. Conf. Comput. Inf. Big Data Appl. (2024)	86.47	84.44
MDFN Cai et al. (16)	Biomedical Signal Processing and Control (2024)	80.78	89.19
FOC-Net Li et al. (42)	Biomedical Signal Processing and Control (2025)	85.37	92.06
Proposed EGP-Net	–	**88.32**	**92.65**

Where ↑ represents the advantage of a higher metric. With the best-performance metrics highlighted in bold for clarity and emphasis.

#### 5.1.2 Performance on the MID-FAHGMU dataset

To better validate the superiority of the proposed model, experiments were conducted on the MID-FAHGMU private dataset. As shown in **Table 9**, EGP-Net exhibited superior performance on multiple standard evaluation metrics, notably surpassing most commonly used comparison methods in IoU (65.25%), Dice (77.46%), F2-score (79.48%), and F0.5-score (77.66%). Compared with existing approaches such as U-Net, Attention U-Net, UNet3+, M2SNet, TransUNet, and SwinUnet, EGP-Net demonstrates consistent and robust improvements in most metrics, particularly in segmentation overlap and detection sensitivity-related measures. Although Attention U-Net achieved the best performance on the F0.5-score, indicating that this method places greater emphasis on improving segmentation precision under this metric, EGP-Net still maintains a more balanced performance across all evaluation criteria. Compared with the results on public datasets, the overall segmentation performance of all methods on this private dataset shows a significant decline. This is mainly attributed to the substantial interference caused by complex lung parenchymal textures, as well as the predominance of extremely small pulmonary nodules in the dataset. Nevertheless, the proposed method still outperforms other segmentation approaches in this highly challenging task, demonstrating its competitiveness and robustness.

**Table 9:** Quantitative evaluation of lung nodule segmentation results using four metrics on the MID-FAHGMU private dataset, where ↑ indicates that higher metric values are better.

MID-FAHGM					
Method	Source	Evaluation metrics			
		IoU (%)↑	Dice (%)↑	F2-score (%)↑	F0.5-score (%)↑
U-Net Ronneberger et al. (12)	MICCAI	63.36	76.06	76.27	77.13
Attention U-Net Schlemper et al. (35)	MIA	64.57	77.31	77.49	79.33
UNet3+ Huang et al. (36)	ICASSP	64.68	77.19	77.99	77.50
SAR-U-net Wang et al. (37)	CMPB	62.60	75.46	74.34	76.73
Swin-Unet Cao et al. (38)	ECCV	52.04	60.57	60.41	63.80
UCTransNet Wang et al. (15)	AAAI	61.98	74.62	74.87	76.78
M2SNet Zhao et al. (39)	TMI	55.08	68.08	68.91	69.86
TransUNet Chen et al. (17)	MIA	60.29	73.47	72.41	76.75
DA-TransUNet Sun et al. (18)	FIBB	61.12	73.95	73.93	76.41
BRAU-Net++ Lan et al. (20)	IEEE	62.00	75.09	75.90	76.23
EGP-Net	Our	**65.25**	**77.46**	**79.48**	**77.66**

With the best-performance metrics highlighted in bold for clarity and emphasis.

**Figure 9:**
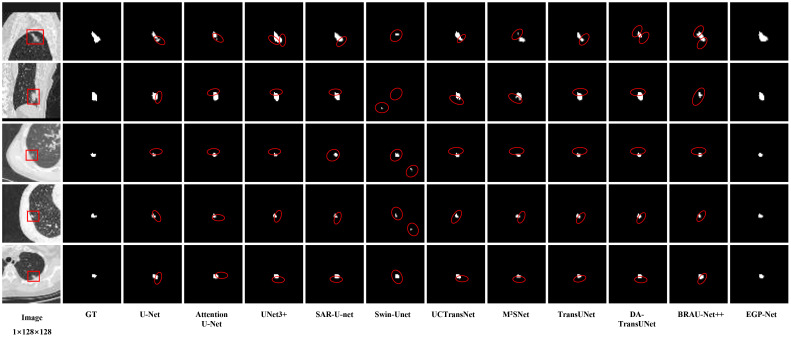
Visualization results of multiple methods on the MID-FAHGMU dataset.

**Figure 9** presents representative segmentation results on challenging cases from the MIDFAHGMU dataset. In rows 2, 3, and 5, the nodules are characterized by small size, indistinct boundaries, and low contrast with surrounding tissues, posing substantial challenges for segmentation. In row 2, U-Net exhibits under- or over-segmentation, Attention U-Net produces imprecise contours, UNet3+ and SAR-U-Net show boundary deviations, Swin-Unet and BRAUNet++ suffer from localization errors, UCTransNet and M2SNet inadequately preserve nodule morphology, TransUNet exhibits shape distortion, and DA-TransUNet generates relatively blurred boundaries. By contrast, EGP-Net achieves more precise boundary delineation and better morphological preservation. Similar observations can be made in row 3, where U-Net undersegments the target region, UNet3+ produces indistinct contours, and Swin-Unet fails to localize the nodule accurately, whereas EGP-Net preserves both nodule shape and boundary information more effectively. In row 5, many methods struggle to distinguish the small nodule from adjacent tissues, resulting in incomplete or inaccurate segmentation. In comparison, EGP-Net produces more complete segmentation results with clearer boundary definition. These visual comparisons are consistent with the quantitative results and further demonstrate the effectiveness of EGP-Net in handling small nodules with blurred boundaries and low contrast.

In rows 1 and 4, the presence of large nodules, irregular morphology, and indistinct boundaries poses challenges for several methods. U-Net, Attention U-Net, M2SNet, and DA-TransUNet show limited detail preservation, Swin-Unet exhibits segmentation errors, and UNet3+, SAR-U-Net, and UCTransNet tend to oversegment the target regions. In contrast, EGP-Net more effectively separates nodules from surrounding tissues and preserves both boundary details and structural integrity. The slight discrepancy observed in row 4 may be attributed to annotation uncertainty associated with very small nodules in the MID-FAHGMU dataset. In addition, the relatively limited dataset size (1,610 CT slices) further increases segmentation difficulty. Nevertheless, EGP-Net maintains consistent segmentation performance across nodules with varying sizes, morphologies, and levels of complexity.

### 5.2 Complexity analysis

**Table 10** reports the computational cost and model size of each method. As shown in the table, EGP-Net is not the most computationally efficient model. This is mainly because the proposed model reconstructs skip connections to effectively guide multi-scale feature fusion, thereby introducing additional feature interactions and computational overhead. In addition, EGP-Net adopts a relatively deep network architecture to enhance its capability to model complex spatial contextual information and fine-grained structures, which is effective for improving the segmentation accuracy of boundary regions and small target regions in medical images. Although these design choices result in higher computational requirements and a larger model size than some competing methods, they contribute to superior segmentation accuracy.

**Table 10:** Computational cost comparison of different methods.

Method	U-Net	Attention U-Net	UNet3+	SAR-U-Net	Swin-U-Net	UCTransNet	M2UNet	TransUNet	DA-TransUNet	BRAU-UNet++	EGP-Net (ours)
FLOPs (G)	13.67	16.64	49.92	17.13	22.72	10.80	2.25	8.05	8.29	5.59	20.76
Model Size (MB)	118.42	133.05	102.88	136.89	157.71	252.69	105.64	355.65	360.52	193.64	158.54

### 5.3 Limitations

**Figure 10:**
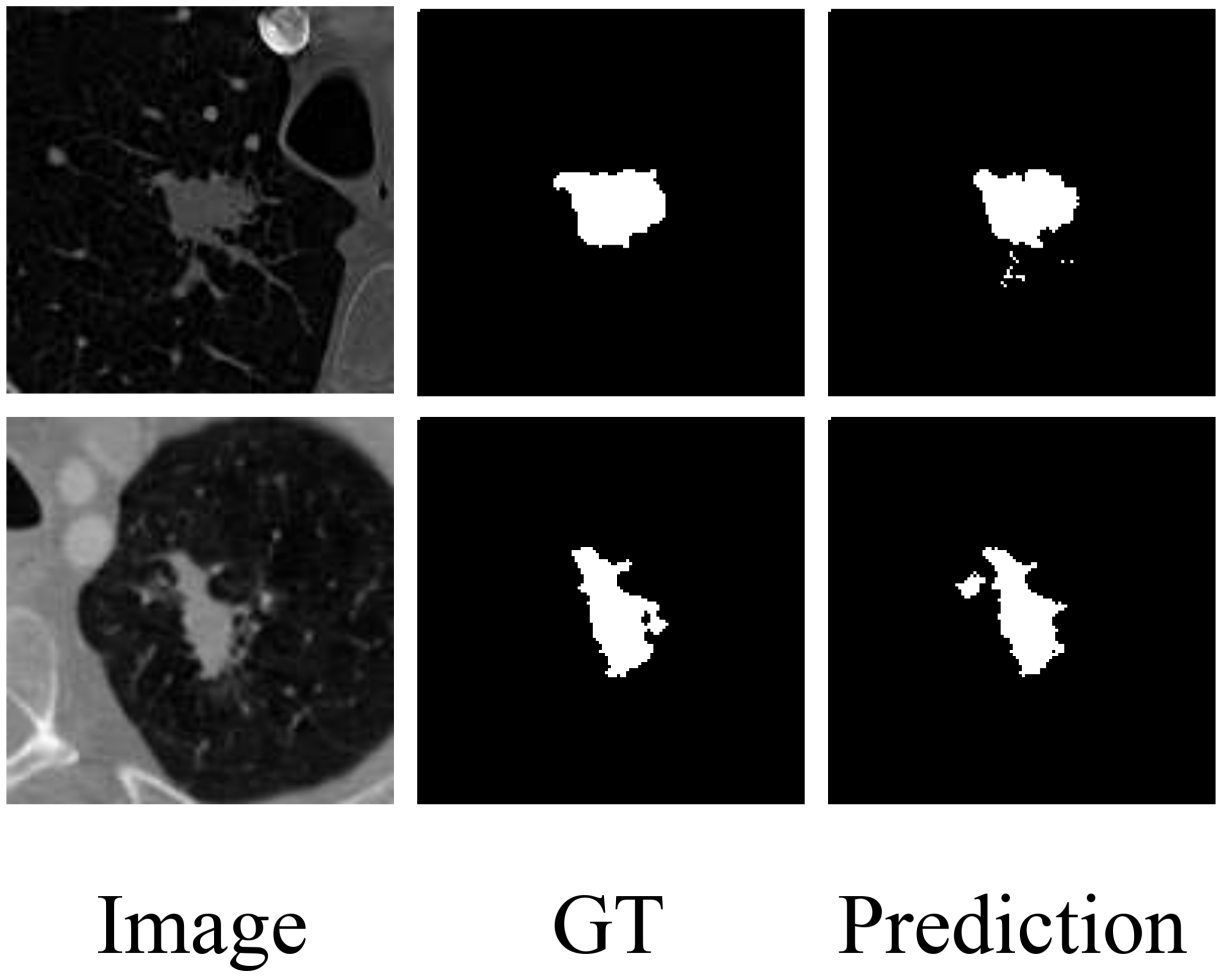
Examples of EGP-Net segmentation failure.

These above analyses demonstrate that the proposed EGP-Net demonstrates certain advantages and strong robustness in the task of pulmonary nodule segmentation. However, EGP-Net fails to successfully segment some challenging scenarios, such as ground-glass nodules with low-density contrast and heterogeneous nodules with complex internal structures. **Figure 10** illustrates several failure cases of EGP-Net in pulmonary nodule segmentation. As shown, the upper-row ground-glass nodules have low contrast with the surrounding lung parenchyma and blurred boundaries, which allows the model to roughly segment the region but makes it difficult to capture precise boundary details. The lower-row solid nodules exhibit more complex characteristics, including multiple small cavities or lucent areas, uneven density, and irregular lobulated edges. Such internal heterogeneity and complex boundary features increase segmentation difficulty and ultimately affect the model’s performance.

### 5.4 Future work

To further expand the clinical applicability of EGP-Net, future work will focus on the following key research directions. First, it is critical to strike a balance between inference speed and segmentation accuracy to meet practical clinical requirements. Accordingly, this study intends to further investigate compact network designs as reported in Bruschi et al. (19) to cut redundant feature computation and elevate the deployment efficiency of the model. Second, to boost the model’s recognition performance for subtle structures, we intend to adopt post-processing optimization strategies Carletti et al. (43) for improving segmentation accuracy and clinical applicability regarding complex nodules. Third, most existing segmentation models are built upon 2D CT images and cannot fully exploit inter-slice spatial correlations embedded in 3D volumetric data. Future research will explore three-dimensional segmentation architectures to improve the model’s ability to characterize the three-dimensional morphology of nodules and their spatial interactions with adjacent tissues. Meanwhile, volumetric metrics including relative volume error will be added to the evaluation system to comprehensively assess segmentation performance in terms of nodule volume prediction accuracy.

## 6 Conclusion

This paper proposes a lung nodule segmentation network that integrates edge guidance and a pyramid-based multi-scale contextual attention mechanism, named EGP-Net. The framework incorporates an encoder network, an edge-guided network, a Global Pyramid Perception (GPP) module, a Dynamic Attention Fusion (DAF) module, and a multi-scale context decoder network, which significantly enhances the capabilities of multi-scale feature extraction and feature recalibration, while simultaneously improving the performance of effectively capturing and accurately delineating edge information. Experimental results on the LIDC and MID-FAHGMU datasets show that EGP-Net achieves superior segmentation performance compared with existing methods, highlighting the potential of the proposed approach for lung nodule segmentation. Comprehensive ablation studies further confirm the effectiveness of its core components. 

## Data Availability

The data presented in the study are deposited in the GitHub repository and is publicly available at: https://github.com/1dengli/EGP_Net.
